# Probing Transition-Metal Silicides as PGM-Free Catalysts for Hydrogen Oxidation and Evolution in Acidic Medium

**DOI:** 10.3390/ma10060661

**Published:** 2017-06-16

**Authors:** Thomas Mittermeier, Pankaj Madkikar, Xiaodong Wang, Hubert A. Gasteiger, Michele Piana

**Affiliations:** 1 Department of Chemistry and Catalysis Research Center, Technical University of Munich, Lichtenbergstr. 4, D-85748 Garching, Germany; pankaj.madkikar@tum.de (P.M.); xiaodong.wang@matthey.com (X.W.); hubert.gasteiger@tum.de (H.A.G.); michele.piana@tum.de (M.P.); 2 Johnson Matthey Catalysts (Germany) GmbH, Bahnhofstr. 43, D-96257 Redwitz, Germany

**Keywords:** silicides, electro-catalysts, PGM-free, hydrogen electro-oxidation, hydrogen evolution reaction

## Abstract

In this experimental study, we investigate various transition-metal silicides as platinum-group-metal-(PGM)-free electrocatalysts for the hydrogen oxidation reaction (HOR), and for the hydrogen evolution reaction (HER) in acidic environment for the first time. Using cyclic voltammetry in 0.1 M HClO_4_, we first demonstrate that the tested materials exhibit sufficient stability against dissolution in the relevant potential window. Further, we determine the HOR and HER activities for Mo, W, Ta, Ni and Mo-Ni silicides in rotating disk electrode experiments. In conclusion, for the HOR only Ni_2_Si shows limited activity, and the HER activity of the investigated silicides is considerably lower compared to other PGM-free HER catalysts reported in the literature.

## 1. Introduction

The hydrogen oxidation/evolution reaction (HOR/HER) is one of the most-studied reactions in aqueous and proton-exchange-membrane-(PEM)-relevant electrocatalysis, with platinum (Pt) exhibiting an exchange current density as high as $\approx 0.2\;A/{\mathrm{cm}}_{\mathrm{metal}}^{2}$ [1]. With this high HOR activity, the Pt demand in typical PEM fuel cells (PEMFCs) is mainly dominated by the cathode electrode requirements rather than those of the anode, in order to minimize kinetic voltage penalties due to the orders of magnitude slower oxygen reduction reaction (ORR) [2]. Consequently, only few attempts have been made to substitute Pt with a platinum-group-metal-(PGM)-free catalyst for the HOR, while future targets as low as 0.05 mg_Pt_/cm^2^ for the ORR electrode may render the HOR Pt demand no longer negligible [3]. So far, only compounds of nickel, tungsten and molybdenum demonstrated electrocatalytic HOR activity [4,5]. On most PGMs, both HOR and HER were shown to be mechanistically closely related yielding comparably symmetric HOR and HER activities [6]. However, on non-noble metals, it is commonly observed that they have reasonable HER kinetics while the HOR seems to be hindered by oxide formation. As for the HOR in an acidic environment, the high HER activity of Pt has driven only little demand for the development of PGM-free HER electrocatalysts. To our knowledge, only PGM-free materials based on Ni, W, Mo, and/or Co have exhibited significant activity for the HER. Apart from bare non-noble metals [7,8,9,10,11] that intrinsically suffer from poor acid stability, few types of materials were investigated, amongst those are transition metal carbides [12,13,14,15,16,17], sulfides [18,19,20,21,22,23,24], phosphides [25,26,27], and borides [9,28]. Bi-metallic catalysts from Ni and Co have demonstrated promising activities in alkaline environment [29,30,31], and outstanding, yet experimentally questionable (due to the use of platinum counter electrodes, with possible contamination of the working electrodes), exchange current densities i_0_ of about 1–3 mA/cm^2^ in acid [7,8]. However, silicide compounds of the previously mentioned transition metals have never been studied before for HOR or HER, while being promising in terms of stability against anodic dissolution [32].

In this study, we report the examination of transition metal silicides in terms of their electrochemical behavior in an acidic environment at inert atmosphere, and for the first time, with respect to their HOR/HER properties. Therefore, we first discuss the preparation of various transition-metal silicides and their characterization with X-ray diffraction (XRD) to investigate phase purity. The prepared materials are then characterized electrochemically using cyclic voltammetry in 0.1 M HClO_4_, first to test their stability against dissolution in acid in the relevant potential window, then to determine their HOR and HER activities. As the PGM-free silicides tested in this study exhibit minor or no HOR activity, probing the methanol oxidation reaction (MOR) or other anodic reactions on these materials is not further focused. With respect to the HER, we find minor activity on the investigated silicides compared to literature data available for other PGM-free materials.

## 2. Results and Discussion

**Figure 1:**
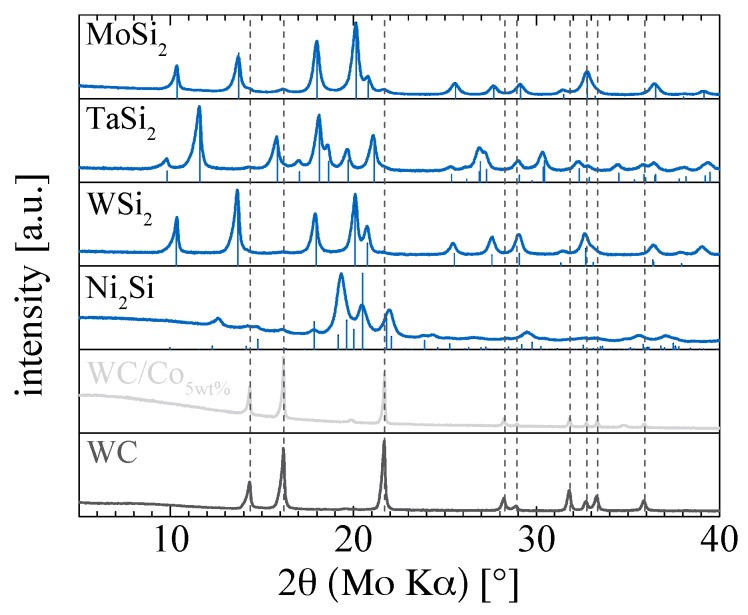
X-ray diffraction patterns of the materials investigated in this study as obtained with a Mo Kα X-ray source. Drop lines indicate reference patterns for MoSi_2_: PDF 410612, TaSi_2_: PDF 380483, WSi_2_: PDF 741149, Ni_2_Si: PDF 768258; the vertical dashed drop lines indicate the WC reference pattern: PDF 510939.

X-ray diffractograms are shown in Figure 1. The diffraction patterns of all samples are well consistent with the reference data of the corresponding silicide phases. The tested silicides are phase pure, with the main reflexes being attributable to the respective material reference. However, the prominent reflexes of tungsten carbide (WC) at 2θ ≈ 14°, 16° and 22° cannot be excluded in the spectra of all produced silicides, which may indicate traces of fragmented ball milling vessel and bead material transferred to these materials. A quantitative assessment of such scored WC from ball milling vessel and beads by energy-dispersive X-ray spectroscopy (EDS) against a 1 wt % WC reference yields values below the detection limit for the silicides tested in this study.

**Figure 2:**
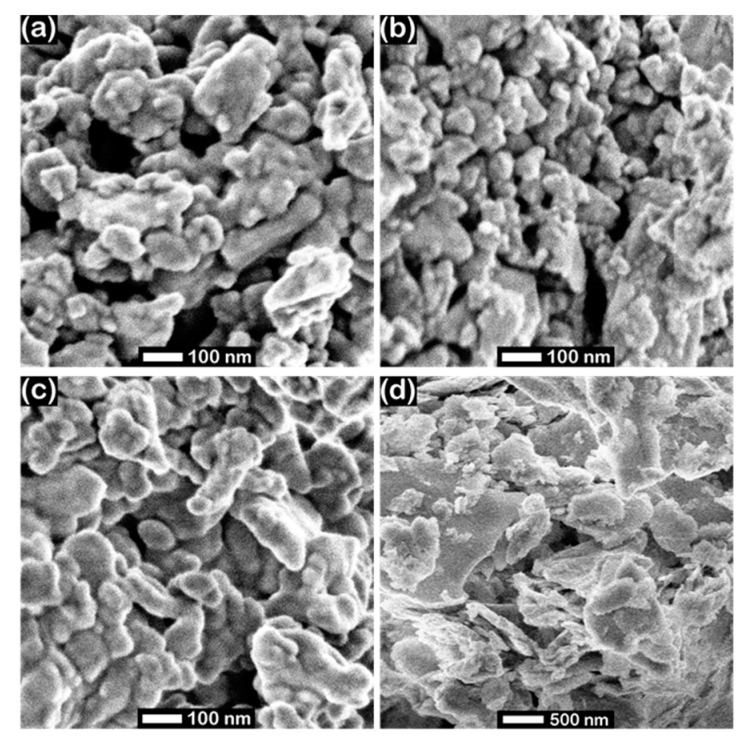
SEM micrographs of the investigated transition metal silicides after ball milling: (**a**) MoSi_2_; (**b**) WSi_2_; (**c**) TaSi_2_; (**d**) Ni_2_Si recorded at 15 kV acceleration voltage.

Brunauer–Emmett–Teller (BET) analyses show that the ball-milled silicides have specific surface areas ranging from 5 to 20 m^2^/g (Table 1). When compared to the metal silicides, WC and WC_5 wt % Co_ have a ≈ 2.5–10 times lower BET surface area (A_BET_). However, it should be noted that all BET surface areas measured on the present samples are at least an order of magnitude lower compared to current state-of-the-art carbon-supported platinum catalysts used in acidic HOR/HER electrocatalysis [1]. From A_BET_, together with the material density ρ_20 °C_ given in Table 1, it is possible to estimate particle diameters of the investigated materials. Assuming spherical shape, we obtain diameters between ≈40 nm (TaSi) and ≈190 nm (WC). It should be noted that this might serve only as a rule-of-thumb estimate, due to the assumption that the particles are spherical and non-porous (both are best-guess estimates from SEM images; see Figure 2). For each of the catalysts, the so estimated particle diameter is several times the value obtained from X-ray diffractograms via the Scherrer equation (see Table 1), suggesting that the samples consist of agglomerates of crystallites (primary particles). This is supported by laser scattering analysis, where the observed number-averaged diameters are between a factor of ≈2–85 higher than those obtained from XRD. Thus, in all cases, agglomerates of primary particles are present on the investigated materials.

Figure 2 shows exemplary scanning electron microscopy (SEM) micrographs of the investigated ball-milled silicides. In case of MoSi_2_, WSi_2_ and TaSi_2_ (a–c), the absence of sharp edges justifies the previously described rule-of-thumb estimate assuming spherical particles for an estimate of particle diameter from BET surface. In the case of Ni_2_Si (Figure 2d), the particles are significantly larger, which is well reflected by the measured number averaged particle diameter obtained via laser scattering (Table 1). In general, the relative trends of particle sizes determined by laser scattering analysis and via SEM exhibit an analogous behavior in this semi-quantitative analysis.

**Figure 3:**
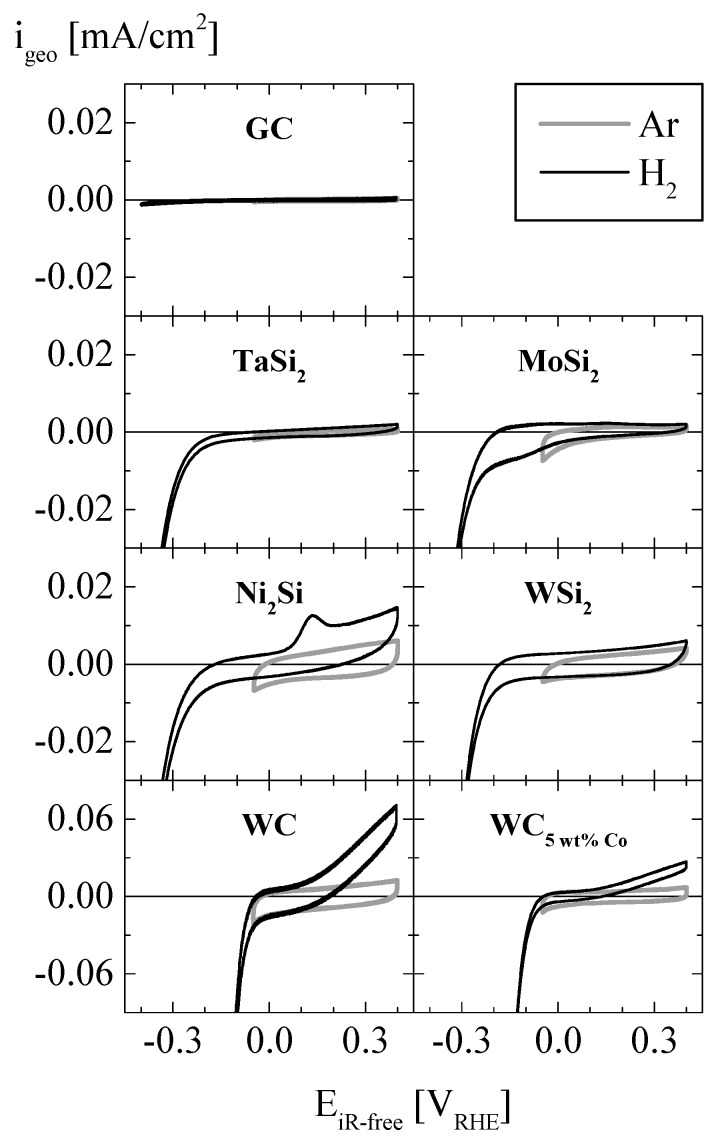
Cyclic voltammograms in 0.1 M HClO_4_, taken at room temperature and a scan rate of 10 mV/s in either Ar (0 rpm, light grey) or H_2_ atmosphere (1600 rpm, black). Catalyst loading for MoSi_2_, TaSi_2_, WSi_2_, Ni_2_Si, WC_5 wt % Co_, WC: 0.50 mg/cm^2^; blank glassy carbon disk (GC) as reference.

Cyclic voltammograms (CVs) of the investigated silicides, carbides, and of a blank glassy carbon (GC) disk as reference in Ar (from 0.05 V_RHE_ to 0.40 V_RHE_) and in H_2_ atmosphere (−0.40 V_RHE_ to 0.40 V_RHE_) are shown in Figure 3. The blank GC does not exhibit any oxidative or reductive features in the applied potential region, as would be expected. MoSi_2_, TaSi_2_, and WSi_2_ exhibit considerable reduction currents below ≈−0.2 V_RHE_, indicating activity towards the HER. At potentials positive of 0 V_RHE_, however, these materials do not exhibit oxidative currents significantly higher than the purely capacitive currents recorded in Ar atmosphere, thus, the hydrogen oxidation reaction (HOR) is not catalyzed at potentials E being 0 V_RHE_ < E < 0.4 V_RHE_. In contrast, Ni_2_Si, WC, and WC_5 wt % Co_ feature oxidative currents at E > 0 V_RHE_, which indicates electrocatalytic activity of these materials towards the HOR. However, both stability limitations (oxidation of materials) and real-life-application considerations (high polarization of fuel-cell anode is not feasible) prevent from opening the potential window more positively, where potential HOR currents would be sufficiently high for quantitative kinetic analyses. Ni_2_Si exhibits an additional oxidation wave at ≈0.13 V_RHE_, with a magnitude negatively correlated with rotation rate and occurring only when the negative potential vertex is <−0.1 V_RHE_ (not shown). With the reduction potential of the Ni^2+^|Ni redox couple situated at −0.19 V_RHE_ at pH = 1, this additional oxidation wave on Ni_2_Si can likely be attributed to the oxidation of underpotentially deposited Ni, a hint that Ni can be leached out of Ni_2_Si and re-deposited when the potential is cycled between low enough values (here <−0.1 V_RHE_) and 0.4 V_RHE_. Since the discussed oxidative wave at ≈0.13 V_RHE_ occurs only when the negative potential vertex is below −0.1 V_RHE_, but also in its absence oxidative currents are observed above 0 V_RHE_, it is likely that anodic dissolution and HOR on Ni_2_Si occur simultaneously at positive potentials upon potential cycling. Further tests on Ni_2_Si and WC_5 wt % Co_ introducing methanol to the electrolyte solution were carried out, however, at no significant activity towards the MOR (not shown). 

**Figure 4:**
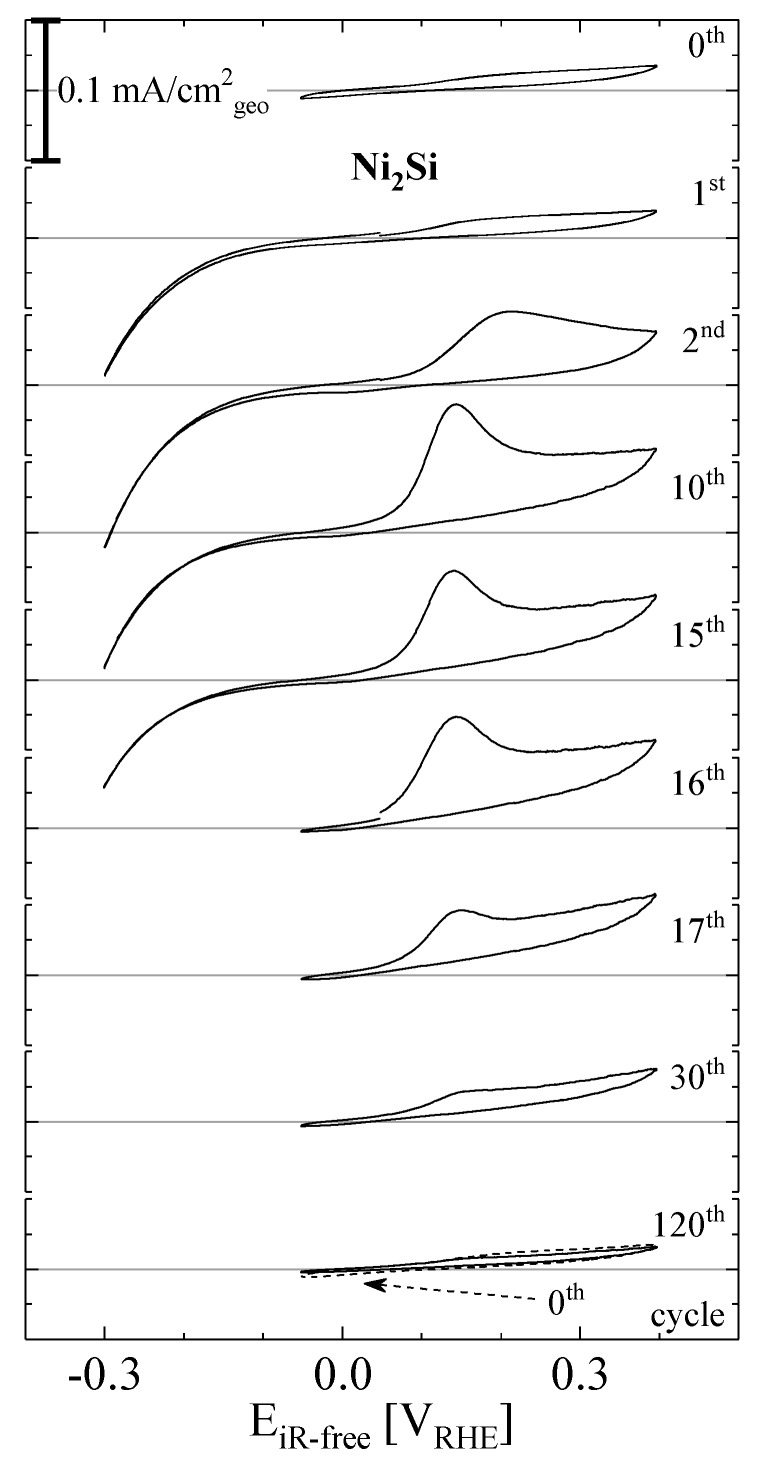
Subsequent cyclic voltammograms of Ni_2_Si in 0.1 M HClO_4_, taken at room temperature and a scan rate of 20 mV/s in H_2_ atmosphere at 1600 rpm. The dashed line in the bottom panel is the same signal as shown in the top panel.

In order to further investigate the origin of the oxidative wave observed on Ni_2_Si at ≈0.13 V_RHE_ (Figure 3), Figure 4 shows a series of Ni_2_Si CVs recorded in an independent experiment, varying the potential window in hydrogen atmosphere at a fixed rotation rate of 1600 rpm. The topmost panel (labeled 0^th^ cycle), shows a steady-state CV of Ni_2_Si in the potential window of −0.05–0.40 V_RHE_, where maximum anodic currents are on the order of ≈0.02 mA/cm^2^, significantly below the diffusion-limited current of ≈3 mA/cm^2^ observed in aqueous electrolyte at comparable conditions [1]. Thus, kinetic losses on Ni_2_Si are dominant against mass-transport for the HOR in the applied potential range, indicating a low HOR activity. Opening the potential window to −0.30 V_RHE_, the 1^st^ CV results in a significant reductive current, and in the subsequent positive going scan (2^nd^ scan in Figure 4) an oxidative wave appears at ≈0.13 V_RHE_ together with significantly increased overall oxidative current at potentials more positive of that. Upon continued potential cycling (10^th^ cycle), a maximum height of the observed oxidative wave is reached, followed by decreasing magnitude of both oxidative (at E > 0 V_RHE_) and reductive (at E < 0 V_RHE_) currents (15^th^). When subsequent CVs are recorded in the previous potential range from −0.05–0.40 V_RHE_ (16^th^–120^th^), both oxidative wave and overall anodic currents decrease and fall even below the values recorded in the 0^th^ scan (for reference indicated as dashed line in the bottom panel of Figure 4). It is noteworthy that the integrated charge (i.e., the area enclosed by the CV) of the 120^th^ scan is over 40% lower than the one observed in the 0^th^ CV, which indicates the loss of a significant amount of surface area during the conducted CVs. 

As hypothesized before (see discussion of Figure 3), the above described behavior of Ni_2_Si can potentially be attributed to a partial reduction and subsequent anodic dissolution of Ni, which would be in line with a mass-loss of Ni_2_Si during potential cycles and finally manifest in a surface area loss as it is indeed observed (see Figure 4). However, a potential second reason to cause an oxidative wave with preceding negative potential excursions would be the formation and storage of hydrogen during excursions to potentials <<0 V_RHE_ either inside the Ni_2_Si compound (absorption) or in the electrode layer (as gas bubbles). In gas phase experiments, Morozkin et al. reported that absorption of hydrogen into similar materials, i.e., lanthanum and cerium nickel silicides is possible [38]. As stated before, the magnitude of the observed oxidation wave at ≈0.13 V_RHE_ gets smaller when rotation rate is increased. Therefore, an absorption of hydrogen into the silicide material seems rather unlikely as a governing mechanism, since it can be expected to be independent of rotation rate in the H_2_ saturated electrolyte. While we indeed cannot rule out either dissolution of Ni or hydrogen accumulation inside the electrode layer with the present results, the occurring mechanism obviously leads to a dramatic loss of catalyst surface, rendering a cathodic activation of Ni_2_Si in acidic environment impracticable.

**Figure 5:**
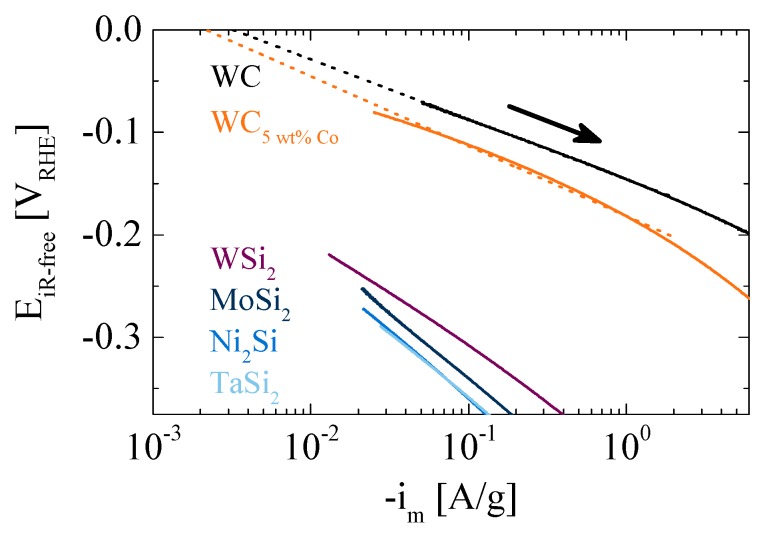
Tafel plot as determined from negative going scans in Figure 3, corrected for capacitive currents obtained from cyclic voltammograms in Ar at 0.05 V_RHE_ (full lines) and linear extrapolations (dashed lines). Extrapolations are performed from an interval where measured current is between three times the capacitive current and 2 A/g.

From Figure 5 is apparent that WC and WC_5 wt % Co_ possess the highest HER activity among the tested materials. Upon investigating sulfide compounds, Bonde et al. [19] state that Co_2_S lacks stability and de-activates upon subsequent HER polarization scans. They also find a promotion of the HER activity by Co addition to WS. This finding from sulfide compounds, obviously, cannot directly be translated to WC/WC_5 wt % Co_ investigated here, where WC_5 wt % Co_ exhibits no superior HER activity compared to pure WC. It is noteworthy that the latter two materials are comparable to the materials utilized in the ball-mill vessel and beads to produce nanometric powders. However, scoring of such ball-milling material does not lead to high erroneous activities on the produced nanometric silicides in this study, as on WSi_2_, MoSi_2_, Ni_2_Si, and TaSi_2_, no measureable amounts of WC could be found by EDS analysis (see discussion of Figure 1). 

In order to enable a quantitative assessment of the HER activity of the tested materials, exchange current densities are extracted via a Tafel extrapolation. To do so, measured data from negative potential scans (cf. Figure 3) are corrected by capacitive current. The capacitive currents for the relevant WC and WC_5 wt % Co_ samples are 0.025 and 0.012 A/g, respectively (estimated from the currents obtained under Ar atmosphere at 0.05 V_RHE_; cf. Figure 3). Figure 5, showing the so corrected currents, however, reveals non-linear trends, rendering a precise estimation of the exchange current density difficult. It is noteworthy that at HER currents close to and beyond the diffusion limit of ≈3 mA/cm^2^ (although determined for the HOR, this transport-related limit should be somewhat significant for HER as well [1]) evolved gaseous H_2_ would shield parts of the electrode. Bearing in mind possible limitations stemming from capacitive currents and from accumulated H_2_, we choose a window between 0.025/0.049 A/g for WC_5 wt % Co_/WC (corresponding to ≈3 times the capacitive current) and 2 A/g (corresponding to 1/3 of the diffusion limiting current) for the fits. The resulting Tafel slopes are 68/58 mV/decade for WC_5 wt % Co_/WC. An analogous evaluation of the currents obtained on WSi_2_ yields a slightly higher Tafel slope of 107 mV/decade. Since its activity is considerably lower than the one observed on WC and WC_5 wt % Co_, an extrapolation to 0 V_RHE_ over roughly two orders of magnitude can be considered an order-of-magnitude-estimation, with a resulting exchange current density of ≈1 × 10^−4^ A/g (corresponding to ≈1 × 10^−9^ A/cm^2^, normalized to BET area).

It is noteworthy that we implicitly assume any reduction of current observed on the investigated materials to originate from hydrogen evolution in the discussion above. For the WC based materials investigated here, this can be considered a valid assumption, since WC has been reported to exhibit profound activity for the HER before [5,39]. For the investigated silicide materials, we observe only limited activities, and therefore omit the proof that reductive current stems only from the HER instead of any possible parasitic reduction in this place, while stringently it would be necessary to prove in any potential application for the HER electrocatalysis. 

Mass based exchange current densities i_0_^m^ of WC and WC_5 wt % Co_ investigated in this study are of comparable magnitude as reported for Mo-based carbides (Table 2). In contrast, WC exhibits almost one order of magnitude higher i_0_^m^ compared to the value reported by Xiao et al. [11] (3 vs. 0.53 mA/g). Classifying this discrepancy remains speculative, as the authors do not provide an estimation for the active surface area of their sample. One possible reason would be a bigger particle size, thus a lower geometric surface area. The same argument holds true for Esposito and co-workers, who report a geometric exchange current density of 2.7 µA/cm^2^ for a WC foil, pre-etched upon potential cycling to the oxidative onset potential [12,13]. One may note that a roughness factor of ≈15 (not unlikely for a pre-etched foil) would be sufficient to explain the discrepancy between their data and the data reported on nanometric WC (or WC_5 wt % Co_) in this study (cf. Table 2).

The previously described results indicate no significant activity of the investigated silicide materials for the HOR and limited activity for the HER. Previous studies on noble metals [6,41] have demonstrated activation energies for the HOR/HER of ≈15–30 kJ/mol (roughly equivalent with a factor of ≈3.5–7 between the exchange current densities when increasing the temperature from 25 °C to 80 °C). While it is beyond the scope of the present study to perform a detailed investigation on the effective activation energies as function of pH, as done by Durst et al. [6] and by Sheng et al. [41], it should be noted that a significant increase of the HER/HOR activity of transition metal silicides at elevated temperatures would require a comparably high activation energy.

## 3. Materials and Methods 

### 3.1. Chemicals

Tantalum disilicide (TaSi_2_), tungsten silicide (WSi_2_, 99.5%) and nickel silicide (Ni_2_Si, 99%, 0.1%–1% of Co) were purchased from Alfa Aesar (via Thermo Fisher (Kandel) GmbH, Karlsruhe, Germany). Molybdenum disilicide (MoSi_2_, ≥99%) was obtained from Aldrich (Sigma, Darmstadt, Germany). Tungsten carbide nanopowder with and without addition of cobalt (WC, 99.95%, 30–100 nm, hexagonal and WC_5 wt % Co_, 99.9%, 40–80 nm, hexagonal) was bought from US Research Nanomaterials, Inc. (Houston, TX, USA). All organic solvents and other chemicals were of analytical grade and used as received.

### 3.2. Preparation of Metal Silicide Nanopowders with Ball Milling

The commercial metal silicide raw materials were ground by wet high-energy ball milling (Fritsch Planetary Pulverisette 7 Premium Line, Fritsch GmbH, Idar-Oberstein, Germany, WC-Co (6 wt %) vessel (20 mL) and balls (diameter of 10, 3 and 0.6 mm)) with 1-octadecene as the dispersion medium. For grinding MoSi_2_ and WSi_2_ powders (both with particle size ≤20 μm), around 1 g of the silicide was mixed with 5–8 mL of 1-octadecene and 30 g of balls (Ø of 0.6 mm) in the vessel. The milling was conducted at 400 rpm for 4 h. In the case of TaSi_2_ (particle size ≤50 μm) and Ni_2_Si (up to 500 μm), 1 g of the powder dispersed in 3 mL of 1-octadecene was first ground at 800 rpm for 2 h with balls of 3 mm (30 g, for TaSi_2_) and 10 mm (80 g, for Ni_2_Si). Afterwards, the milled powder was further ground with balls of 0.6 mm at conditions described above. A grinding‒pausing time interval of 2–10 min was applied to avoid significant increase of temperature and pressure in the system. After milling, the resulting powders were isolated by centrifugation, washed with hexane, and finally dried at ambient conditions.

### 3.3. Characterization of the Metal Silicides Catalysts

The particle size of metal silicides was measured with a laser scattering particle size distribution analyzer (HORIBA Scientific LA-950V2, HORIBA, Ltd., Kyoto, Japan) using ethanol as dispersing medium. BET analyses were carried out using an automated gas sorption analyzer (autosorb^®^iQ, Quantachrome Instruments, Boynton Beach, FL, USA) to obtain the specific surface area of the powders. X-ray diffraction (XRD) patterns of the silicides were obtained on a STOE powder diffractometer (Stadi MP, STOE & CIE GmbH, Darmstadt, Germany) equipped with a Mo Kα X-ray source. The measurements were performed in transmission mode, where the silicide powder was configured to a thin layer on the sample holder. For scanning electron microscopy (SEM) and energy-dispersive X-ray spectroscopy (EDS), a JSM-6000 benchtop scanning electron microscope (JEOL, Tokyo, Japan) equipped with MP-00040EDAP was used at an acceleration voltage of 15 kV and a working distance of 19 mm at 100-fold magnification. The solid powdered samples were transferred on a copper tape (3M) with adhesive conductive coating for the measurements.

In order to prepare electrodes for cyclic voltammetry, the catalyst powder of interest was suspended in 70/30 vol % H_2_O/2-propanol and 2 × 10^−5^ g_Nafion_/m^2^_BET_ were added via 5 wt % Nafion^®^ solution (Sigma, Darmstadt, Germany). The ink was dispersed in an ice-cold ultrasonic bath for 30 min, before pipetting 10 µL onto a freshly polished glassy carbon rotating disk electrode (5 mm diameter, Pine Research Instrumentation), resulting in a catalyst loading of 500 µg/cm^2^. The electrochemical set-up employed is reported in a previous publication, using a gold mesh in a glass-frit separated compartment (25 × 25 mm^2^, 82 mesh, 0.060 mm wire diameter, 99.99% purity, Advent Research Materials, Eynsham, UK) as the counter electrode and a trapped hydrogen electrode separated with an electrolyte bridge (Pt wire of 1.0 mm diameter sealed into a glass plug with end drawn to a capillary, >99.99% purity, Advent Research Materials, Eynsham, UK) as the reference electrode [42]. All cyclic voltammograms were recorded in 0.1 M HClO_4_, at room temperature and the indicated rotation rate. The electrolyte was prepared from a 60% stock solution (Guaranteed Reagent, Kanto Chemical Co., Inc., Tokyo, Japan) by dilution in ultrapure water.

## 4. Conclusions 

This study reports probing of transition metal silicides for the HER and HOR for the first time. The single-metal silicides investigated here do not exhibit profound activity for the HER and HOR in acidic electrolyte. On WC, we demonstrate both mass- and surface-specific exchange current densities at ≈2–3 mA/g and ≈0.1–0.2 µA/cm^2^, reasonably consistent with values reported in literature, taking into account that most cited references do not provide sufficient information about the active surface area of their materials (a task admittedly difficult at unknown reactive centers).

## Figures and Tables

**Table 1:** Physico-chemical properties of the materials tested in this study. Particle size as determined by different techniques; *Number*: by number-averaged laser scattering analysis; *XRD*: by broadness of XRD reflections using Scherrer equation; *BET*: via A_BET_ assuming spherical particles (i.e., 6/(ρ_20 °C_*A_BET_)). A_BET_ indicates the surface area based on Brunauer–Emmett–Teller experiments, σ_20 °C_ and ρ_20 °C_ are the electrical conductivity and density at 20 °C, respectively.

Material	Size [nm]			A_BET_ [m²/g]	σ_20 °C_ [kS/cm]	ρ_20 °C_ [g/cm³]
	Number	XRD	BET			
MoSi_2_	138	13	48	20	5.9–7.9 [33]	6.3 [34]
TaSi_2_	79	11	37	18	2.5–5.0 [33]	9.1 [34]
WSi_2_	129	15	61	10	7.8–8.4 [33]	9.9 [34]
Ni_2_Si	865	10	152	5	4.2 [35]	7.9 [34]
WC_5 wt % Co_	76	42	≈192	2	--	≈15.6 [36]
WC	313	22	192	2	7.0 [37]	15.6 [36]

**Table 2:** HER activities of PGM-free catalysts demonstrated in this study (t. s.) in comparison to reported materials. The asterisk (*) marks an estimation by the authors of the present study. The surface area used to calculate i_0_^s^ was determined as indicated: CV irrev. Ox. means the quantification of anodic charge as explained by the authors of the cited study, STM is scanning tunneling microscopy, H_upd_ means the integration of hydrogen ad-/desorption charge in a cyclic voltammogram in inert atmosphere and, where stated, the determined BET area was used.

Material	Electrolyte	i_0_^m^ [mA/g]	Surface Area Via	i_0_^s^ [µA/cm²]	Ref.
WC_5 wt % Co_	0.1 M HClO_4_	2	BET	0.11	t. s.
WC	0.1 M HClO_4_	3	BET	0.16	t. s.
WC	0.5 M H_2_SO_4_	0.53	--	--	[11]
Mo_2_C	0.5 M H_2_SO_4_	4.5	--	--	[11]
Mo_2_C/CNT	0.1 M HClO_4_	6.9	--	0.046	[14]
MoS_2_/C	0.5 M H_2_SO_4_	--	CV irrev. Ox.	1.2	[19]
[Mo_3_S_4_]^4+^/HOPG	0.5 M H_2_SO_4_	--	STM (rf = 1 *)	0.22	[40]
Pt/C	PEMFC	2.6 ± 0.7 × 10^8^	H_upd_	2.2 ± 0.5 × 10^5^	[1]
